# Supplementary material for: Patterns of Intron Gain and Loss in Fungi
Source: PLoS Biol. 2004 Nov 30;2(12):e422. doi: 10.1371/journal.pbio.0020422 (PMC532390; doi:10.1371/journal.pbio.0020422)
Supplement: Table S1 — Also available at http://genes.mit.edu/NielsenEtAl/. (4.3 MB ZIP). [file pbio.0020422.st001.zip › NielsenEtAl/html/1230.html]

AN8869.1.NCU01446.1.MG06915.1.FG07475.1


```
 CLUSTAL W (1.82) Multiple Sequence Alignments - Introns Inserted


Sequence 1: NCU01446.1	244 aa
Sequence 2: MG06915.1	275 aa
Sequence 3: FG07475.1	256 aa
Sequence 4: AN8869.1	249 aa
Alignment Length: 282 aa
Number Identitical Residues: 166 aa
Alignment Score (without introns) 7183


MG06915.1 	-----MPAAKPPS--QGVGPVYQAEHQKPTATVSTDVTRFANVTVLPQTPQLIALLT2II
NCU01446.1	---MSTASEIAPT--KCVGPDYRAEEQKPTATVSTAVN-FDNVTVLSQTPQLIALLS2II
FG07475.1 	---MADKIQDSHT--TPVGPAYRTHQQKPSATVSVDVN-LDNVHVLPQTPQLIALLS2MI
AN8869.1  	MSVMSGPAEPIPSPAQEVGPVYRSDRGKPTATVSKSIS-HENVHVLPQTPQLIALLT2MI
          	 :  :       :.:  *** *::.. **:****  :.   ** **.*********: :*

MG06915.1 	RDKATERGDFIFYSNRIIRLLVEEGLNHLPTVEHTVTTPVGRPYAGLMFQGKICGVSIMR
NCU01446.1	RDKRTDRGDFIFYSNRIIRLLVEEGLNHLPTVEHPVTTPVGRIYDGLAFQGKICGVSIMR
FG07475.1 	RSKETERADFIFYSNRIIRLLVEEGLNHLPVIEHTVTTPIGRTYNGLMFQGKICGVSIMR
AN8869.1  	RDQNTGRADFIFYSNRIIRLLVEEGLNHLPVVENSVTTPVGRSYLGVKFEGKICGVSIMR
          	*.: * *.**********************.:*:.****:** * *: *:**********

MG06915.1 	AGEAMEQGLRDCCRSVRIGKILIQRDEETATPKLFYDKLPEDIASRWVLLLDPMFATGQF
NCU01446.1	AGEAMEQGLRDCCRSVRIGKILIQRDEETAQPKLFYDKLPEDIADRWVLLLDPMFAT---
FG07475.1 	AGEAMEQGLRDCCRSVRIGKILIQRDEETAQPKLFYDKLPEDIADRWVLLLDPMFATGMT
AN8869.1  	AGEAMEQGLRDCCRSVRIGKILIQRDEETCMPKLFYEKLPTDISNRWVLLLDPMFAT---
          	*****************************. *****:*** **:.************   

MG06915.1 	GQRSTPCPSSWASTPQRVALGSNMPLRVSG~GSAIMAVEVLKGRGVPEERILFLNLIASP
NCU01446.1	-----------------------------G1GSAIMAVEVLKSRGVPEERIIFLNILASP
FG07475.1 	TSRHVP--------------------QTAR1GSATMAVQVLKARGVPEEHILFLNLIASP
AN8869.1  	-----------------------------G1GSATLAVETLKAKGVPEERILFLNLIASP
          	                               *** :**:.**.:*****:*:***::***

MG06915.1 	EGIENFATKFPKLRVVTAFVDQ~GLDEKN2YIIPGLGDFGDRFYTM
NCU01446.1	EGISNFASKFPKLRTVTAFVDQ~GLDDKN2YIIPGLGDFGDRYYSM
FG07475.1 	EGVKNFSAKFPRLRVVTAFIDE0GLDEKN2YIVPGLGDFGDRFYTI
AN8869.1  	SGVADFAQRFPKLRVVTAFIDQ~GLDEKK2YIIPGLGDFGDRYYTL
          	.*: :*: :**:**.****:*: ***:*: **:*********:*::
```
